# Experimental investigation on the reverse mechano-electrical effect of porcine articular cartilage

**DOI:** 10.3389/fbioe.2025.1485593

**Published:** 2025-02-03

**Authors:** Chunsheng Liu, Le Zhao, Hao Dong, Zekun Hua, Yanqin Wang, Yongxing Wang, Pengcui Li, Xiaochun Wei, Kai Zhang, Yanru Xue, Xiaogang Wu, Weiyi Chen

**Affiliations:** ^1^ College of Biomedical Engineering, Taiyuan University of Technology, Taiyuan, China; ^2^ Taiyuan Great Health Technology Health Management Co., Ltd., Taiyuan, China; ^3^ Shanxi Provincial Key Laboratory for Repair of Bone and Soft Tissue Injury, Taiyuan, China; ^4^ Huajin Orthopaedic Hospital, Taiyuan, China

**Keywords:** articular cartilage, reverse mechano-electrical effect, cartilage’s thickness, deflection, external electric field

## Abstract

**Introduction:**

The electric signals within the cartilage tissue are essential to biological systems and play a significant role in cartilage regeneration. Therefore, this study analyzed and investigated the reverse mechano-electrical effect in porcine articular cartilage and its related influencing factors.

**Methods:**

The deflection of cartilage samples in an electric field was measured to analyze the mechanisms of different factors affecting the reverse mechano-electrical effect in articular cartilage.

**Results:**

The results showed that the cartilage thickness, water content, and externally applied voltage all impacted the deflection of the cartilage. The reduction in cartilage water content resulted in a decrease in cartilage thickness, following the same influencing mechanism as thickness. On the other hand, an increase in the externally applied voltage led to an increase in the electric field force within the cartilage space, consequently increasing the deflection of the cartilage in the electric field. Additionally, the externally applied voltage also caused a slight temperature rise in the vicinity of the cartilage specimens, and the magnitude of the temperature increase was proportional to the externally applied voltage.

**Discussion:**

The fitting results of the experimental data indicated that cartilage thickness influenced the dielectric constant and moment of inertia of the cartilage in the electric field, thereby affecting the magnitude of the electric field force and deflection of the cartilage. This may provide valuable insights for further investigation into the microscopic mechanisms of cell proliferation, differentiation, and cartilage regeneration induced by electrical stimulation.

## 1 Introduction

Osteoarthritis is one of the most common diseases among the global adult population, affecting hundreds of millions of people worldwide. Furthermore, its prevalence is expected to continue increasing in the coming years (Glyn-Jones et al., 2015; Disease et al., 2018). Due to the avascular nature of articular cartilage, it cannot naturally regenerate after injury. Therefore, clinical treatment is typically required when damage or disease occurs (Hunziker, 1999). The therapeutic effects of currently used clinical treatment modalities are not entirely satisfactory (Zhou et al., 2022). Therefore, it is necessary to seek a new approach that can effectively stimulate and accelerate cartilage regeneration. In recent years, electrical stimulation (ES) has been widely recognized as a promising method to promote cartilage regeneration. It can facilitate cartilage regeneration by directly applying electrical stimulation or harnessing the endogenous bioelectricity within the cartilage (Hu et al., 2014; Clark et al., 2014). These pathways alter gene expression in the cells and promote the production of growth factors (Leppik et al., 2020). Additionally, electrical fields can stimulate the expression of aggrecan and type II collagen mRNA, as well as increase the production of proteoglycans and collagen in human osteoarthritic cartilage explants (Brighton et al., 2008). Bioelectricity within cartilage tissue stands as an essential component of the biological system. Endogenous electric fields, characterized by weak electrical signals emanating from cartilage during daily physiological activities, play a pivotal role in early embryonic development and tissue regeneration (Kapat et al., 2020). These electrical signals have the potential to impact crucial processes such as cell migration, proliferation, and differentiation within cartilage tissue. Recognizing the significance of bioelectricity, a range of electric stimulation techniques have been devised for clinical applications, encompassing the acceleration of wound healing, deep brain stimulation, and tissue regeneration. Given the pervasive nature of bioelectric signals throughout the body, electrical stimulation emerges as a key strategy in promoting the regeneration of cartilage tissue (Kwon et al., 2016; Curry et al., 2020). In the past few decades, electrical stimulation has gained increasing attention due to its applications in regenerative medicine (Zimmermann et al., 2023). As early as 1972, Becker and Spadaro (1972) discovered that altering wound polarity by applying exogenous electrical stimulation in mammals could improve wound healing and potentially induce tissue regeneration response. Zuzzi et al. (2013) conducted a 35-day electrical stimulation on rat articular cartilage defects. The results revealed that continuous electrical stimulation led to the increased thickness of collagen fibers in the cartilage and a higher number of chondrocytes, indicating a promoting effect of electrical stimulation on articular cartilage repair. Furthermore, Vaca-Gonzalez et al. (2019) demonstrated that electrical stimulation can promote cell proliferation and stimulate the synthesis of matrix molecules associated with articular cartilage cells, such as type II collagen, proteoglycans, and glycosaminoglycans. Despite significant research endeavors, the translation of these findings into clinical applications in the relevant field remains elusive. This challenge can be attributed to the limited scope of conclusions drawn from preclinical *in vitro* and *in vivo* experiments, which may not yield directly applicable insights for clinical implementation. Therefore, it is necessary to consider the effects of exogenous electrical stimulation on the organism itself. Leppik et al. (2015) demonstrated the good tolerance of the organism to long-term direct current electrical stimulation by applying it to a rat amputation model. Moreover, no additional side effects were noted, including weight changes, decreased vitality, signs of infection, or tumor development. This confirmation underscores the effectiveness of electrical stimulation as a therapeutic approach for osteoarthritis.

The cartilage reverse mechano-electrical effect involves the mechanical deformation of the cartilage surface induced by external electrical field stimulation. This phenomenon leads to ion flow and changes in electric potential within the cartilage, thereby influencing its normal physiological functions. Specifically, the mechanical deformation resulting from the reverse mechano-electrical effect triggered by electrical stimulation can alter the local microenvironment at the site of cartilage defects, activate signaling pathways on cell membranes, and regulate the expression of relevant genes to enhance chondrocyte activity and promote cartilage regeneration (Zhang et al., 2014; Wieland et al., 2015; Papachroni et al., 2009). Currently, research on the reverse mechano-electrical effect is mainly focused on cartilage regeneration, including chondrocyte proliferation, differentiation, and migration (Housmans et al., 2023). It utilizes low-intensity direct current electric fields to promote cartilage regeneration, reduce symptoms of arthritis, and thereby improve articular function and restore the articular structure. In recent years, significant progress has been made in the research on the reverse mechano-electrical effect in cartilage. For example, Hiemer et al. (2018) have demonstrated that the direct application of exogenous electrical stimulation to cartilage can effectively mimic the endogenous electric potentials generated within the cartilage tissue during articular motion and mechanical loading processes. This stimulation facilitates the healing process, guides the development of cartilage cells, and supports cartilage tissue regeneration. Consequently, the researchers explored the effects of electric fields on human chondrocytes, mesenchymal stem cells, and co-cultures of both. The outcomes demonstrated that applying electrical stimulation to cartilage did not impact the metabolic activity of chondrocytes or bone marrow mesenchymal stem cells. Moreover, osteoarthritis presents significant challenges for the individual, such as pain and limited mobility. Pelletier et al. (2015) discovered that the pain caused by arthritis is not only a result of pathological changes in the peripheral tissues surrounding the cartilage but also involves sensitization of the central and peripheral nervous systems and a decrease in descending pain inhibition. Additionally, the application of electrical stimulation to cartilage has a Neuromodulation effect. Therefore, electrical stimulation can effectively alleviate the pain caused by Antal et al. (2010). Therefore, delving into the cartilage reverse mechano-electrical effect can offer crucial references for clinical practice to enhance osteoarthritis conditions and advance cartilage tissue regeneration through the application of electrical stimulation to the cartilage.

In 1996, Aschero et al. (1996) experimentally validated the existence of the reverse mechano-electrical effect in fresh bovine bone. The researchers placed cylindrical specimens of bovine bone, measuring approximately 10 mm in thickness, into a specialized dual-chamber extensometer. They applied an electric field with an intensity of 10 kV/m and recorded measurements of bone thickness changes along and across the electric field lines. The results revealed that the bovine bone specimens exhibited a displacement deflection of approximately 3 µm under the influence of the applied high electric field, thus confirming the presence of the reverse mechano-electrical effect in bovine bone tissue. Given the similarities in properties between bone and cartilage, this study suggests employing a similar methodology to conduct experiments targeting articular cartilage. The goal is to explore the potential physical mechanisms underlying the deflection and flexural behavior of cartilage when exposed to electrical stimulation. This will provide valuable insights for further investigation into the microscopic mechanisms of cell proliferation, differentiation, and cartilage regeneration induced by electrical stimulation. Compared to previous studies, we introduced non-contact electric fields for the first time in our experiments, evaluating the characteristics of cartilage by utilizing displacement signals generated through the reverse mechano-electrical effect.

## 2 Materials and methods

### 2.1 Preparation of cartilage specimens

**FIGURE 1:**
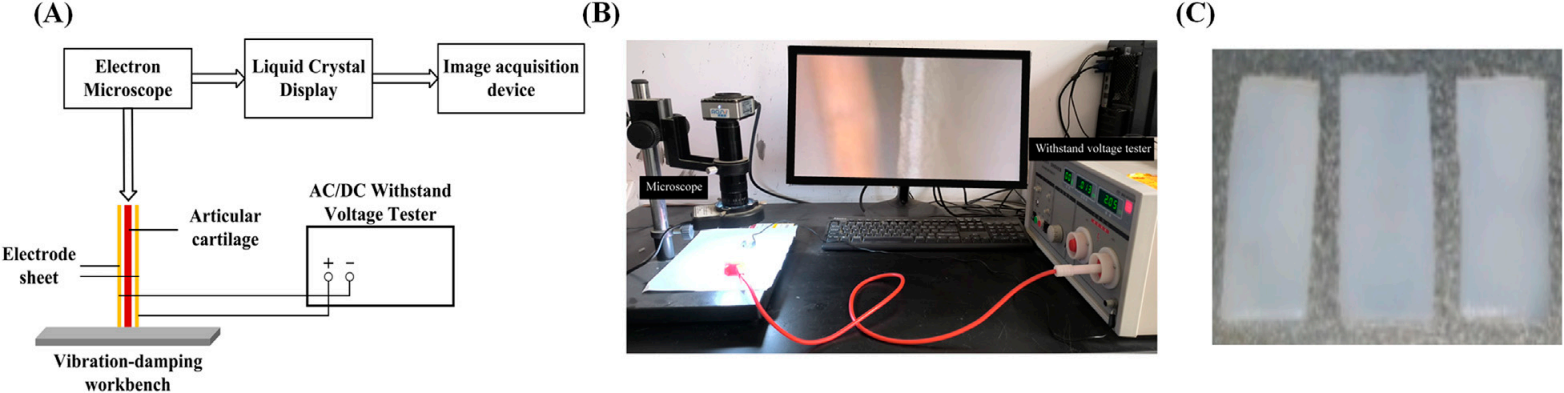
Experimental testing apparatus images **(A)** Schematic image; **(B)** Physical images; **(C)** Sample image.

Shortly after slaughter, the cartilaginous tissue of the pig’s hind leg articular is detached for sampling. The pig meat and cartilage utilized for post-slaughter experimentation do not necessitate approval procedures. Carefully remove the muscles around the cartilage on the clean table, open the joint capsule to expose the cartilage, and then remove the fascia. Observe the cartilage surface, cut the cartilage specimen with a smooth position without slit, and then cut with a scalpel to obtain a 20 mm 10 mm rectangular specimen. The specimen is milky white and smooth in texture, as shown in Figure 1C. After sampling, the specimen thickness was controlled at 1–2 mm by polishing the cartilage on 1,000 target sandpaper. Additionally, to minimize structural discrepancies among specimens, ensure that sandpaper grinding is consistently directed from bottom to top, thereby minimizing variations across different regions of the specimen.

### 2.2 Reverse mechano-electrical effect testing device

The experimental setup, as shown in Figure 1, includes a high-pressure endurance testing device, a high-magnification industrial microscope, a computer monitor, a cartilage fixation device, and two copper electrode plates. First, fix the prepared cartilage specimen onto the fixation device. Then, utilize the external high-pressure endurance testing device to apply different direct-current fields. The electron microscope (AO-HD228S) captures the deflection and flexure of the non-fixed end of the cartilage specimen and transmits these changes in real-time to the computer monitor. By selecting appropriate observation points in the monitor and pre-calibrating them, the deflection and flexure of the cartilage can be determined. To study the reverse mechano-electrical effect, we measured the deflection and flexure of pig articular cartilage under the influence of an electric field. The experimental samples were obtained from the articular cartilage of pig joints, specifically from the articular surface. The experiments were conducted using samples of varying thickness: 1, 1.2, 1.4, 1.6, 1.8, and 2.0 mm (all with a length and width of 10 mm). The articular cartilage specimens were placed within an external electric field generated by two electrode plates connected to a high-voltage steady-state direct current power supply (LK2674 Pressure Tester). The deflection and flexure of the free end of the cartilage specimens were observed and calculated using a high-magnification electron microscope and digital image processing techniques. This allowed us to explore various properties of the reverse mechano-electrical effect in cartilage. Before the experiment, the cartilage samples were soaked in 0.9% physiological saline solution, and the surface of the cartilage was wiped to remove any excess moisture before the experiment commenced.

## 3 Results

### 3.1 The effect of different thicknesses on cartilage deflection deformation

**FIGURE 2:**
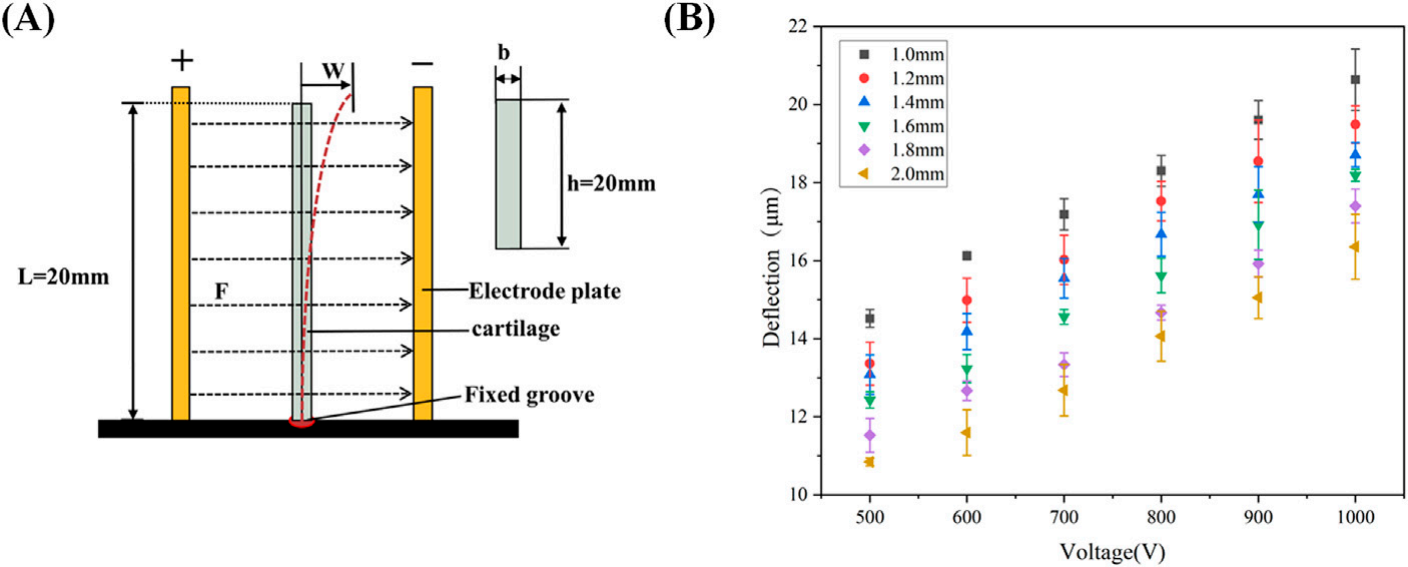
**(A)**: Schematic diagram of cartilage displacement in an electric field; **(B)** Deflection of cartilage of different thicknesses under different voltages.

Figure 2A presents a schematic diagram illustrating the deflection of the cartilage specimen within an electric field, showing how the cartilage bends and flexes in response to the applied electric field force. In Figure 2B, the magnitude of deflection and flexure induced by the reverse mechano-electrical effect in cartilage is depicted under varying thicknesses (1, 1.2, 1.4, 1.6, 1.8, and 2.0 mm) when an external electric field is applied. The applied voltage is maintained at 600 V, and the water content is maintained between 75% and 80%. For example, with an applied voltage of 600 V, as the cartilage thickness decreases from 2.0 mm to 1.0 mm, the deflection and flexure within the electric field increase from 11.7 μm to 16.2 μm. This trend holds across different voltage groups, indicating that a reduction in cartilage thickness leads to a gradual increase in deflection and flexure within the electric field, demonstrating an inverse relationship between these variables.

### 3.2 The effect of different moisture content on cartilage deflection deformation

**FIGURE 3:**
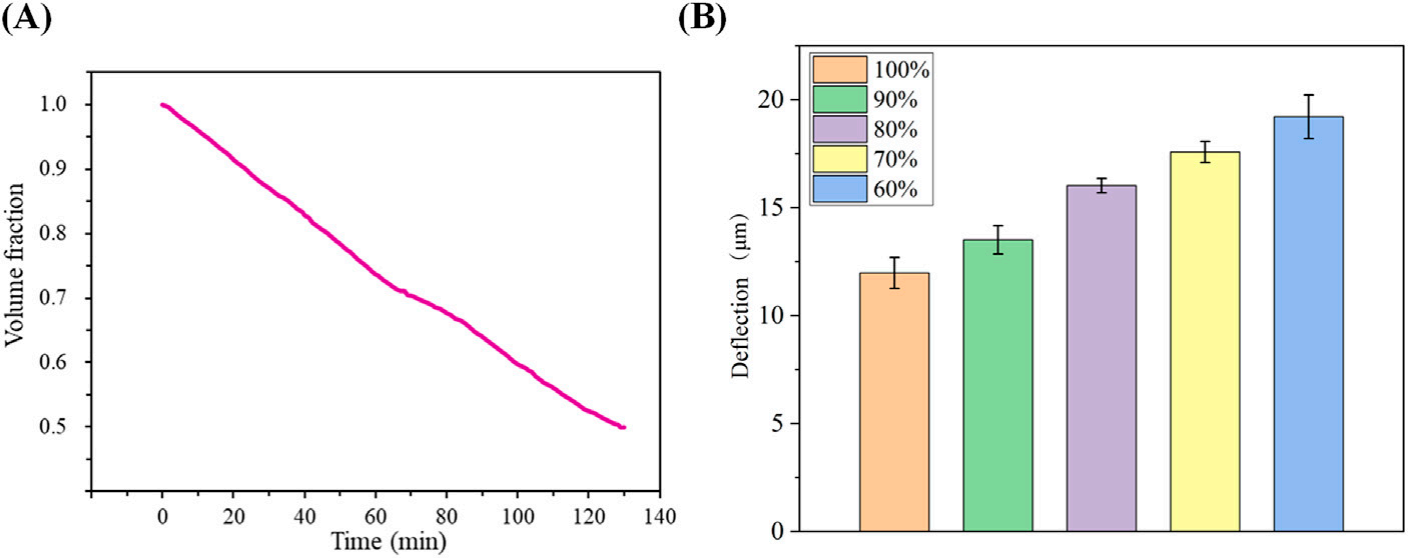
**(A)**: Average dehydration of cartilage over time at room temperature; **(B)** Magnitude of cartilage deflection due to different water contents.

The fitted curve in Figure 3A illustrates the average dehydration rate of the cartilage specimens at room temperature. It indicates that it takes approximately 129.5 min for the cartilage to lose water from a saturated state (after soaking in physiological saline for 24 h) to reach 50% water content. This fitting result also suggests that the dehydration rate of the cartilage specimens under room temperature conditions follows a linear change. The dehydration rate of the sample volume fraction is approximately 24% per hour. In Figure 3B, the deflection displacement generated by cartilage at a thickness of 1.0 mm under an applied voltage of 600 V is shown for different water contents. The results reveal that as the water content gradually decreases, the deflection displacement of the cartilage in the electric field increases from 13.9 μm at water saturation to 17.4 μm at 60% water content. This indicates an inverse relationship between the deflection displacement in the electric field and the water content of the articular cartilage under the same external conditions.

### 3.3 The effect of different voltages on cartilage deflection deformation

**FIGURE 4:**
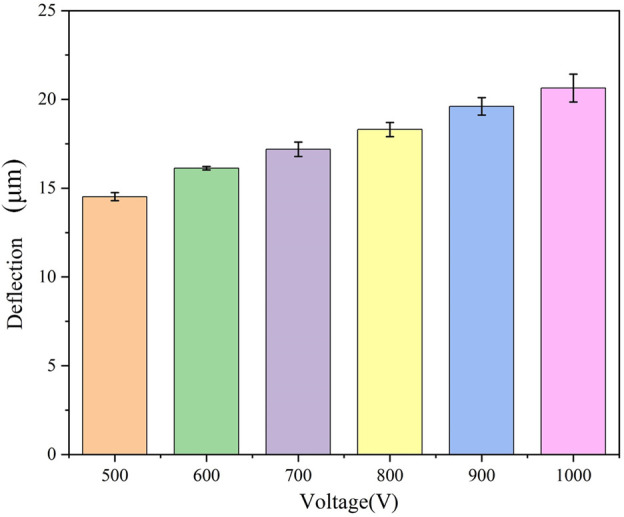
Magnitude of cartilage deflection produced under different voltages.

We conducted tests to examine the effect of various applied external electric fields (500, 600, 700, 800, 900, and 1000 V) on the deflection displacement generated by cartilage due to the reverse mechano-electrical effect replaced, while maintaining a constant cartilage thickness (1.0 mm) and water content range (75%–80%). The experimental findings, illustrated in Figure 4, demonstrate that the magnitude of the applied external electric field notably influences the deflection displacement resulting from the inverse piezoelectric effect in cartilage. When using an applied external electric field of 500 V, the measured deflection displacement of the cartilage was 14.3 μm. As the applied external electric field gradually increased, the deflection displacement of the cartilage specimen also showed an increase. At an external electric field reached 1000 V, the corresponding deflection displacement of the cartilage rose to 20.2 μm. Hence, it can be concluded that increasing the applied external voltage results in a proportional increase in the deflection displacement generated by the reverse mechano-electrical effect in cartilage, indicating a significant positive correlation between the two factors.

### 3.4 The effect of external voltages on cartilage temperature variation

**FIGURE 5:**
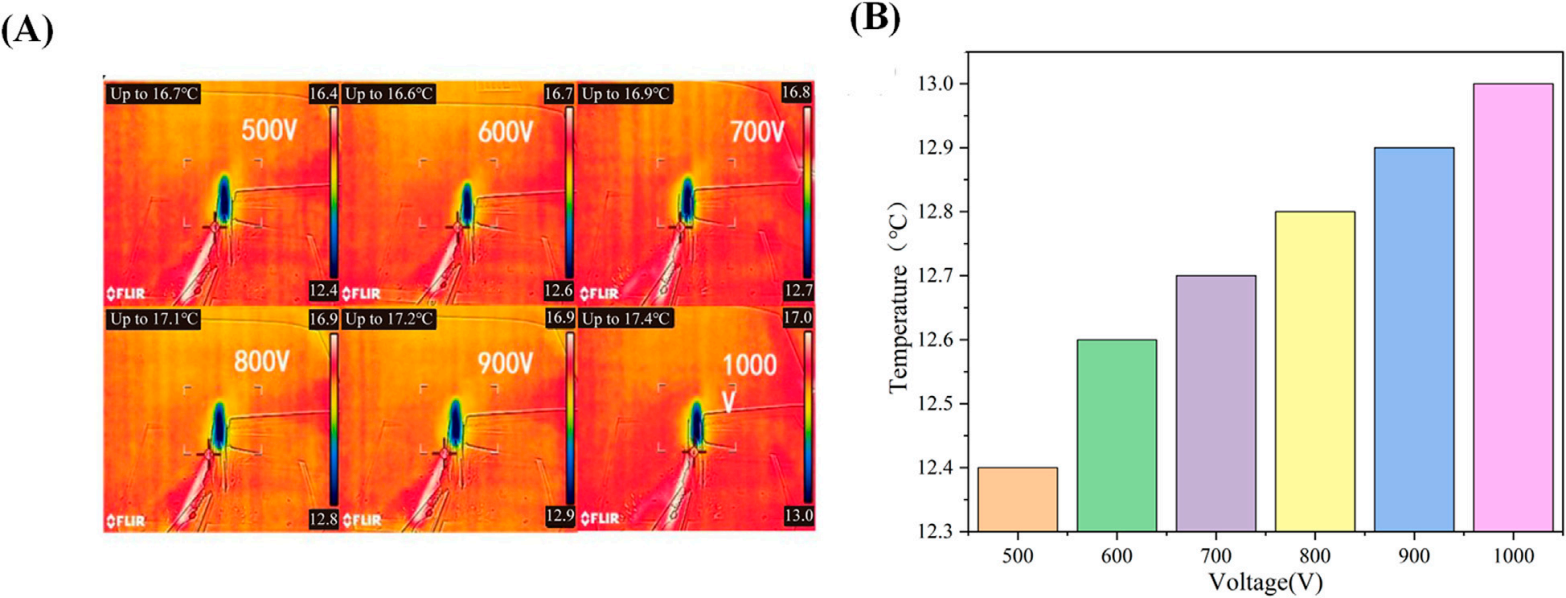
**(A)**: Infrared thermal imaging of cartilage under different voltages; **(B)** Surface temperature of cartilage under different voltages.

Due to the influence of the water content of the cartilage on the specimen’s thickness during the experiment, changes in water content are significantly affected by the external temperature. Therefore, we conducted tests to measure the temperature variations of the cartilage specimen itself and its surrounding environment at various applied voltages (500, 600, 700, 800, 900, and 1000 V). Figure 5A presents the infrared thermal images of the cartilage and electrodes at different applied voltages. As the cartilage specimens had been soaked in physiological saline for 24 h, it is evident that the temperature of the cartilage is significantly lower than the ambient temperature. Upon applying the external voltage, there is a slight increase in the temperature around the cartilage. As the applied voltage increases, the temperature around the cartilage also rises. The corresponding results are depicted in Figure 5B. The relationship between the two can be approximated as an increase of 0.1°C increase in the temperature around the cartilage for every 100 V increase in the applied external voltage.

## 4 Discussion

The magnitude of deflection displacement exhibited by articular cartilage in an electric field is a result of both the inherent structural characteristics of the cartilage itself and the external loading conditions. Macroscopic studies focusing on the reverse mechano-electrical effect in cartilage offer valuable insights into various physiological processes, such as cell proliferation and differentiation within cartilage, which are triggered by microscopic-level electrical stimulation signals. In our study, we conducted a comprehensive examination of the deflection displacement induced by the inverse piezoelectric effect in pig articular cartilage when subjected to an electric field. We thoroughly analyzed how cartilage dimensions and external conditions influence the extent of deflection displacement, while also taking into account the impact of external voltage on the surface temperature of the cartilage. Through meticulous analysis and fitting of experimental data, we discovered that the thickness of the cartilage plays a crucial role in determining the dielectric constant and moment of inertia of the cartilage within the electric field. These factors, in turn, influence the magnitude of electric field forces and resulting deflection displacement in the cartilage.

With the advancement of biomedical technology and tissue engineering, electrical stimulation for cartilage regeneration has gained increasing attention. Numerous studies have robustly demonstrated that electrical stimulation can effectively enhance the self-healing capacity of cartilage and promote cartilage tissue regeneration (Zhou et al., 2023; Krueger et al., 2021; Vaiciuleviciute et al., 2023). However, the direct application of high-voltage external electrical stimulation to cartilage tissue can pose a risk of damage to the organism itself (Victoria et al., 2009). Therefore, it is crucial to find a suitable approach that is sensitive to electrical stimulation signals to promote cartilage repair while minimizing potential damage to the organism itself. In recent years, with the rise of tissue engineering research, the use of implantation of alternative materials to promote cartilage repair has become a reliable clinical treatment approach (Liu et al., 2022). This technique heavily relies on the selection of implant materials. Given the beneficial effects of electrical stimulation on cartilage repair, piezoelectric materials that are sensitive to electrical stimulation signals appear to be a promising choice. These materials can mimic the microenvironment within cartilage and generate stimulating biological responses. Additionally, they can generate electrical signals themselves to promote tissue repair. Therefore, combining the reverse mechano-electrical effect with biomaterials for articular research seems to be an effective approach to enhance and promote cartilage regeneration. Consequently, we conducted research on the mechanisms and related influencing factors of the reverse mechano-electrical effect in cartilage.

The study indicates that there is a significant correlation between the deflection displacement of cartilage in an electric field and the moment of inertia of the cartilage itself, as well as the magnitude of the electric field force applied externally. The specific relationship between these factors can be described as in Equation 1 (Philpot, 2008):

$$w=-\frac{\frac{F}{L}L^{4}}{8E_{p}I_{y}}$$ (1)

w represents the deflection displacement of cartilage in the electric field, F represents the magnitude of the electric field force applied to the cartilage specimen, L represents the length of the cartilage specimen, Ep represents the elastic modulus of the cartilage specimen, and Iy represents the moment of inertia of the cartilage.

In the experiment, we measured the average elastic modulus of the cartilage specimen with a thickness of 1.0 mm as 6.13 MPa. Therefore, we only need to investigate the relationship between the cartilage thickness and the electric field force F and moment of inertia Iy. Since the cartilage specimen is placed between two electrode plates, we can approximate the experimental setup as a parallel plate capacitor. Thus, the electric field force can be described as follows Equation 2:

F=Q·E (2)

Q represents the charge between the two electrode plates, and E represents the electric field intensity. The relationship between Q and E can be described as in Equations 3, 4:

Q=C·U (3)

$$E=\frac{U}{d}$$ (4)

C represents the capacitance, U represents the magnitude of the applied voltage, and d represents the distance between the two electrode plates. The capacitance C is defined as in Equation 5:

$$C=\frac{\varepsilon S}{4\pi kd}$$ (5)

where: ε represents the dielectric constant of the cartilage, S represents the relative area between the two electrode plates, and k represents the electrostatic force constant. Based on the above equations, we can conclude Equation 6:

$$F=\frac{\varepsilon SU^{2}}{4\pi kd^{2}}$$ (6)

According to the definition of the moment of inertia, it can be described as follows:

$$I_{y}=\frac{\;bh^{3}}{12}$$ (7)

where: b represents the thickness of the cartilage, and h represents the width of the cartilage specimen. Therefore, we obtain the relationship between the deflection value of cartilage in the electric field and the thickness of the cartilage as follows:

$$w=-\frac{3\varepsilon SU^{2}L^{3}}{8{\pi kd^{2}bE}_{p}h^{3}}$$ (8)

From Equations 7, 8, it is evident that when the thickness b of the cartilage specimen is altered while keeping other conditions constant, the moment of inertia Iy of the cartilage decreases with decreasing thickness b. thereby affecting the deflection displacement w of the cartilage. Additionally, since changes in cartilage thickness result in variations in the cartilage’s dielectric constant ε, the electric field strength between the two electrodes remains constant under the same voltage. However, due to the different thicknesses leading to different dielectric constants ε. the electric field force F experienced by the cartilage becomes inconsistent. The combined effect of these factors significantly influences the deflection of the cartilage induced by the reverse mechano-electrical effect.

**FIGURE 6:**
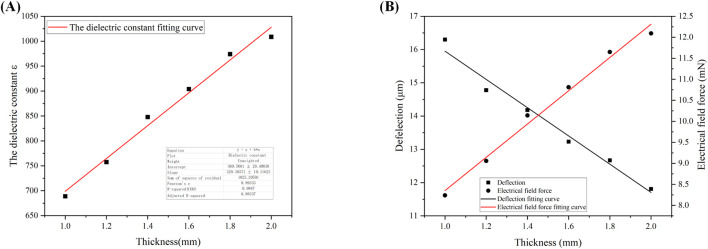
**(A)**: Fitted curve of dielectric constant as a function of cartilage thickness; **(B)** Fitted curves of deflection and electric field force as a function of cartilage thickness.

As we can observe the magnitude of cartilage deflection in the electric field through a high-magnification industrial microscope during the experiment, the magnitude of the electric field force F cannot be directly determined due to the unknown dielectric constant ε. Thus, we can deduce the magnitude of the electric field force from the deflection values, allowing us to explore the relationship between cartilage thickness and the electric field force F as well as the dielectric constant ε. The specific relationship is depicted in Figure 6, with the following external conditions set: the relative area (S) of the two electrodes is 20 mm × 12 mm, the applied voltage (U) is 600 V, the cartilage specimen width (h) is 10 mm, the length (L) is 20 mm, and the distance (d) between the two electrodes is 8 mm. The fitting results indicate a linear relationship between cartilage thickness and the electric field force F, the deflection w as well as the dielectric constant ε. In other words, as the cartilage thickness increases, the electric field force F,the deflection w and the dielectric constant ε also increase. This relationship can be described by the Equations 9–11:

ε=329303.71b+369.51 (9)

$$F=\frac{82325.93bSU^{2}+92.38SU^{2}}{\pi kd^{2}}$$ (10)

$$w=-\frac{123488.89bSU^{2}L^{3}+138.57SU^{2}L^{3}}{{\pi kd^{2}bE}_{p}h^{3}}$$ (11)

The specific interpretation is as follows: When the thickness of the cartilage specimen increases, it becomes more difficult for the specimen to be penetrated, resulting in a larger dielectric constant.

Furthermore, Equation 8 indicates an inverse relationship between the deflection displacement w and the cartilage thickness b. This is consistent with our fitting results for the relationship between cartilage thickness and deflection displacement shown in Equation 11 and Figure 6B, thus validating the reliability of the data obtained in our experiment.

The thickness of the cartilage sample emerges as a critical determinant in our exploration of the reverse mechano-electrical effect, underscoring its pivotal role in our research. To maintain methodological rigor and ensure precision in our comparisons, we systematically tested identical samples with varying thicknesses, progressing from thicker to thinner specimens. Throughout these experiments, we upheld consistency in other influential variables such as cartilage area and the applied external voltage. The experimental findings, elegantly depicted in Figure 2B, unveil a pronounced and noteworthy relationship between cartilage thickness and deflection displacement within the electric field under unwavering external conditions. This observed correlation elegantly mirrors an anticipated negative trajectory, closely aligning with our initial hypotheses. This association can be traced back to the experimental setup where one end of the cartilage is anchored, while changes in specific markers at the opposing end are meticulously tracked post-voltage application. This setup profoundly influences the electric field force acting on the cartilage and the cartilage’s moment of inertia. The thickness of the cartilage profoundly impacts both its dielectric constant and moment of inertia, culminating in a discernible negative correlation between cartilage thickness and deflection displacement in the electric field.

Cartilage is characterized by its high water content, a defining feature (Ansari et al., 2019). However, due to the relatively thin thickness of the cartilage specimens, they tend to lose water rapidly at room temperature. The application of voltage can potentially elevate the surrounding temperature of the cartilage, further expediting the dehydration process. Hence, water content is a crucial factor that cannot be overlooked in the study of the reverse mechano-electrical effect in cartilage. To mitigate this concern, we procured cartilage samples and stored them in physiological saline to ensure adequate hydration during the experiments. Before initiating the experiments, we initially plotted the average dehydration curve of the cartilage samples. The fitted results, as depicted in Figure 3A, indicate that the cartilage dehydration follows a linear trend over time at room temperature. In our study, we examined three sets of cartilage specimens, with individual variations having minimal impact on cartilage dehydration. Therefore, we identified specific points on the dehydration curve and recorded the corresponding time required to reach those points. The impact of water content on the deflection displacement induced by the reverse mechano-electrical effect in cartilage is shown in Figure 3B. As the water content decreases, under consistent conditions of cartilage thickness (1.0 mm) and applied external voltage (600 V), the deflection displacement of the cartilage in the electric field increases. This observation may be attributed to the decreased water content within the cartilage, leading to a thinner cartilage thickness and subsequently influencing the deflection displacement in the electric field. In experiments, it is important to acknowledge the inherent error that may arise from the equipment setup. Firstly, there is a degree of uncertainty in the measurements captured by the high-power microscope, although this error is deemed insignificant relative to the screen resolution. The electric field error introduced by the pressure tester was carefully controlled to be within 1 V, but still had a slight adverse effect on the precision of the experiment. Additionally, the selection of a semi-flexible fixation method for the fixation device was chosen for the convenience of conducting multiple measurements, yet its potential impact on the experiment remains unpredictable.

In the experiment, we observed a significant impact of different applied voltages on the deflection of the cartilage in an electric field. Therefore, we examined the pattern of variation in the reverse mechano-electrical effect in cartilage by adjusting the applied voltage. Figure 4 illustrates the deflection of the cartilage samples at various voltages (500, 600, 700, 800, 900, 1000 V). The results reveal a notable positive correlation between the applied voltage and the deflection of the cartilage, suggesting that higher applied voltages result in increased deflection. This phenomenon can be elucidated as follows: during the experiment, the cartilage specimens are placed between two electrode plates, and upon applying voltage, an electric field is generated between these plates. The electric field exerts a force on the cartilage, causing a corresponding deflection. According to Equation 4, the electric field intensity escalates with increasing applied voltage. With other conditions constant, the electric field force acting on the cartilage also increases, leading to a larger deflection in the electric field. Thus, based on the experimental results, it can be inferred that the applied voltage significantly influences the deflection of cartilage in an electric field, exhibiting a positive correlation between the two.

In light of the high voltage levels utilized in our experimental setup, it is imperative to address the inevitable temperature fluctuations that arise. Consequently, we diligently monitored the temperature variations surrounding the cartilage specimens both before and after the application of varying external voltages. To maintain the reliability of our experimental data, we ensured a period of stabilization, allowing the temperature to return to normal room levels before proceeding with subsequent measurements. The results, elegantly depicted in Figure 5, shed light on the thermal effects induced by the application of voltage on the cartilage specimens. As expected, our findings indicate a noticeable increase in temperature following the voltage application. Noteworthy is the direct correlation observed; as the applied voltage intensifies, so too does the magnitude of temperature elevation in the vicinity of the cartilage samples. Nevertheless, during the voltage increase from 500 V to 1000 V, the temperature rise around the cartilage specimens remains below 1°C, indicating a relatively small temperature increase. Furthermore, we conducted a thorough investigation into the repercussions of these temperature variations on the water content and thickness variability of the cartilage specimens. Strikingly, our findings revealed minimal differences in both water content and thickness when exposed to these temperature fluctuations. Particularly noteworthy is the negligible impact on the thickness of the cartilage specimens (Walker and Madihally, 2015). Based on these compelling results, we can confidently conclude that while the application of voltage does lead to a slight increase in temperature within the cartilage specimens, this temperature rise has negligible implications for the thickness variations of the cartilage under standard room temperature conditions.

Additionally, this study has several limitations. Firstly, the precision of the high-voltage tester during the application of external voltage is limited, leading to a potential error of up to 10 V in the applied voltage. This introduces a slight deviation in the measured deflection values. Secondly, in the sampling process, the cartilage specimens were manually cut and polished, which unavoidably introduces some errors. Consequently, the size of each sample is not precisely identical, with an error of less than 0.02 mm. To mitigate this, we computed the average values to minimize this error. In addition, it is worth noting that high external voltages can cause a slight temperature increase, which has the potential to influence the cartilage’s dielectric constant within the electric field. However, since the temperature difference observed before and after applying the voltage was found to be insignificant, we chose to overlook this particular impact in our study. Furthermore, due to various factors related to both the cartilage itself and the specifics of our testing setup, a period of approximately 30 s is necessary to properly adjust the applied voltage value after securing the cartilage in the testing apparatus. During this period, the cartilage’s water content evaporates to some extent, resulting in a decrease in water content of approximately 2%. These limitations should be taken into consideration when interpreting the results and further research can be conducted to address these limitations and enhance the accuracy of the findings. In previous research, inevitable discrepancies were observed between experimental outcomes and real physiological indicators, influenced by factors such as temperature variations and the intensity of the electric field (Fukada and Yasuda, 2007). Moving forward, we plan to refine our experimental approach by conducting studies in environments that closely replicate physiological conditions. This adjustment aims to mitigate any discrepancies and better align our experimental results with real-world physiological responses.

Electric stimulation plays a crucial role in cartilage repair, with its impact primarily seen in several key areas. Firstly, it promotes the proliferation of chondrocytes, accelerating the regeneration and repair of cartilage tissue. Through targeted electric stimulation, chondrocyte growth and differentiation are activated, facilitating the formation of new cartilage tissue. Additionally, it enhances the synthesis of cartilage matrix by stimulating chondrocytes, leading to increased production of essential components like collagen and chondroitin sulfate, thus supporting the repair and regeneration of cartilage tissue (Fukada and Yasuda, 2007; Halperin et al., 2004).

Furthermore, electric stimulation improves chondrocyte function by boosting metabolic activity, enhancing cell function and biological activity, ultimately contributing to the health and stability of cartilage tissue. By reducing the inflammatory response in damaged areas and alleviating pain and swelling, appropriate electric stimulation facilitates a smoother cartilage repair process. In summary, electric stimulation comprehensively impacts various stages of cartilage repair, including cell proliferation, matrix synthesis, cell function, and inflammation response, ultimately promoting the healing and repair of cartilage damage.

## 5 Conclusion

In summary, this study investigated the reverse mechano-electrical effect in articular cartilage and its related influencing factors. The findings reveal that applying an external electric field leads to corresponding deflection and flexure of the cartilage. This phenomenon was consistently observed throughout the entire experimental process.

(1) The thickness of cartilage serves as a critical factor influencing the deflection and flexure induced by the reverse mechano-electrical effect within the cartilage. As the cartilage thickness decreases, the deflection and flexure resulting from this effect intensify. Our thorough analysis of the data fitting highlights that changes in cartilage thickness directly impact the cartilage’s dielectric constant within the electric field, consequently affecting the magnitude of the electric field force exerted. Moreover, the cartilage’s moment of inertia is intricately linked to its thickness. The synergistic effect of these elements leads to varying degrees of deflection and flexure within the electric field in response to fluctuations in thickness.
(2) The water content within cartilage is a crucial factor that impacts the deflection and flexure induced by the reverse mechano-electrical effect. This is due to the highly hydrated nature of cartilage, where a decrease in water content leads to a gradual reduction in cartilage thickness. Consequently, this reduction in thickness results in an increased deflection and flexure within the electric field.
(3) In our experimental investigations, we conducted separate tests to quantify the degree of cartilage deflection and flexure across different voltage settings. Our analysis underscores the significant impact of the external electric field strength on the deflection and flexure induced by the reverse mechano-electrical effect in cartilage. These variables demonstrate a positive correlation, illustrating that an escalation in the external electric field intensity directly corresponds to an increase in the deflection and flexure experienced by the cartilage.
(4) Due to the application of a high external electric field, it is inevitable that there will be changes in temperature around the cartilage specimen. Our findings reveal that when an external voltage of 500 V is applied, it causes an approximate temperature increase of 0.1°C around the cartilage. With the applied voltage increases to 1000 V, the temperature rises to 0.7°C. Notably, the temperature increase is approximately 0.1°C for every 100 V increase in voltage.

## Data Availability

The original contributions presented in the study are included in the article/supplementary material, further inquiries can be directed to the corresponding authors.
